# The Relationship Between Socio-Demographic and Behavioral Characteristics and Adherence to the Mediterranean Diet: The UniFoodWaste Study Among University Students in Italy

**DOI:** 10.3390/epidemiologia6030053

**Published:** 2025-09-03

**Authors:** Antonio Pinto, Daniele Nucci, Flavia Pennisi, Lorenzo Stacchini, Nicola Veronese, Stefania Maggi, Carlo Signorelli, Vincenzo Baldo, Vincenza Gianfredi

**Affiliations:** 1 Faculty of Medicine, University Vita-Salute San Raffaele, 20132 Milan, Italy; 2 Ph.D. National Program in One Health Approaches to Infectious Diseases and Life Science Research Department of Public Health, Experimental and Forensic Medicine, University of Pavia, 27100 Pavia, Italy; 3 Struttura Semplice Dipartimentale Igiene Alimenti e Nutrizione, Dipartimento di Igiene e Prevenzione Sanitaria, Agenzia di Tutela Della Salute (ATS) Brescia, Via Duca Degli Abruzzi, 15, 25124 Brescia, Italy; 4 Department of Community Healthcare Network, Health District “Toscana ASL Nord Ovest”, Pisa, Via Fleming 1, 56025 Pontedera, Italy; 5 Faculty of Medicine, Saint Camillus International University of Health Sciences, 00131 Rome, Italy; 6 Neuroscience Institute, Aging Branch, National Research Council (CNR), 35127 Padova, Italy; 7 Department of Cardiac Thoracic Vascular Sciences and Public Health, University of Padua, 35128 Padova, Italy

**Keywords:** mediterranean diet, socio-demographic characteristics, behavioral characteristics, students

## Abstract

Background: Adherence to the Mediterranean diet (MD) is associated with improved health outcomes, however limited evidence exists on the socio-demographic and behavioral determinants of MD adherence among university students, a population at risk of developing unhealthy habits during a critical life stage. Methods: A cross-sectional study was conducted among 2697 students (70.6% female) enrolled at a university in Northern Italy. MD adherence was measured using the validated Medi-Lite score. Multivariable logistic and linear regression models were used to identify socio-demographic and behavioral associations with high adherence to the MD (score ≥12). Principal component analysis was performed to explore multivariate patterns across dietary components and participant characteristics. Results: Overall, 25.6% of participants were classified as having high adherence to the MD. Higher adherence was more frequent among women, non-smokers, older students, and those living with their families. Students in health sciences showed greater adherence compared to those in other fields of study. Conversely, frequent users of mobile food ordering applications and smokers were less likely to adhere to the MD. These associations remained consistent after adjusting for age and sex. Conclusions: Adherence to the MD is suboptimal among university students and influenced by socio-demographic and behavioral factors. Targeted interventions should prioritize younger males, smokers, and convenience food users, while promoting sustainability and social support as facilitators of healthier dietary patterns.

## 1. Introduction

Adherence to healthy dietary patterns is a critical determinant of health and disease prevention across the lifespan [1,2]. Among these, the Mediterranean Diet (MD) has garnered extensive recognition for its role in reducing the risk of chronic non-communicable diseases, including cardiovascular disease [3,4], metabolic syndrome [5,6], certain cancers [7,8,9], and neurodegenerative conditions [10,11]. The Global Burden of Disease Study 2019 estimates that dietary risks are among the leading contributors to premature mortality and disability-adjusted life years (DALYs) in Europe, accounting for over 950,000 deaths and 17 million DALYs annually [12]. Inadequate intake of whole grains, fruits, and vegetables, alongside excessive consumption of red meat and sodium, are among the principal dietary factors contributing to this burden [13,14].

MD is characterized by a high intake of plant-based foods (such as fruits, vegetables, legumes, and whole grains), olive oil as the principal source of fat, moderate consumption of fish and dairy products, and limited intake of red meat and processed foods, the MD reflects not only a nutritional model but broader behavioral characteristics that include conviviality, seasonality, and cultural heritage [15,16]. In particular, prospective cohort studies have shown that high adherence to the MD can reduce cardiovascular mortality [17] by up to 30% and all-cause mortality by approximately 20% [18]. Beyond these benefits, adherence to the MD has been associated with reduced inflammation, improved metabolic profile, and lower incidence of chronic diseases [19,20]. In contrast, the Western dietary pattern, typically rich in red and processed meats, refined grains, added sugars, and saturated fats, has been linked to increased risk of obesity, metabolic syndrome, cardiovascular diseases, certain cancers, and all-cause mortality [21].

In recent years, however, there has been a documented decline in adherence to the MD pattern, particularly among younger generations [22], including university students. For instance, the Italian national surveillance system “OKkio alla SALUTE” [23] and the ARIANNA study [24] by the Istituto Superiore di Sanità have documented low levels of dietary adherence among young people, with fewer than 5% achieving optimal MD adherence scores. This trend is of significant public health concern, as dietary behaviors established during emerging adulthood tend to persist into later life, influencing long-term health trajectories [25]. Factors such as increased urbanization [26], academic stress [27], reduced time and skills for food preparation [28], and widespread availability of ultra-processed foods [29] have contributed to the shift away from traditional dietary patterns toward more Westernized diets [21]. University students represent a vulnerable subpopulation undergoing profound life transitions, often including geographical relocation, changes in living arrangements, and shifts in economic and social support structures [30]. These transitions may impact their food choices and overall diet quality [31]. Socio-demographic factors—such as gender, educational level, area of residence, and cohabitation status—have been shown to influence dietary habits, yet evidence on their relationship with MD adherence within this population remains limited and heterogeneous [32]. Regional differences in health indicators and healthcare quality may also influence dietary behaviors [33]. Moreover, behavioral characteristics, such as smoking habits and the use of mobile food ordering applications, may also play an important role in shaping dietary patterns, yet their association with the Mediterranean diet in young adult populations has been less frequently explored [34].

Understanding the socio-demographic correlations with adherence to the MD in university students is essential for designing effective, evidence-based interventions aimed at promoting healthy eating habits and preventing future diet-related diseases. This study investigates the association between selected socio-demographic and behavioral characteristics and adherence to the Mediterranean diet among university students in Italy, as part of the UniFoodWaste project. A secondary aim is to assess the relationship between behavioral characteristics, including the use of food-related digital applications, and adherence to the Mediterranean diet.

## 2. Materials and Methods

### 2.1. Study Design

A cross-sectional study was conducted to examine the associations between socio-demographic factors and Mediterranean Diet adherence among university students in Italy. Data collection took place from July to October 2023. This design was chosen because it allows the estimation of prevalence and the assessment of associations between variables in a large population within a limited time frame, providing a snapshot of real-life patterns and relationships. The primary objective of the study was to estimate the prevalence of adherence to the Mediterranean Diet in this population, while secondary objectives included identifying socio-demographic, behavioral characteristics, and behavioral factors associated with high adherence.

Ethical clearance for the study was obtained from the University of Milan’s Ethics Committee (Approval ID: 71.23. 20 June 2023.), confirming compliance with established ethical standards and the protection of participants’ rights throughout the study process.

### 2.2. Participants

Students were eligible to participate if they were enrolled at the University of Milan, at least 18 years old (as required by the Ethics Committee), residing in Italy during the study period (to ensure ability to read and respond in Italian), and willing to provide informed consent. Participation was voluntary with no incentives provided for completion or participation. The invitation to participate was sent to all enrolled students via the university’s institutional email system, without restrictions on year of study or academic discipline. The sampling aimed to reflect the diversity of university students in terms of age, background, and living arrangements. Exclusion criteria included (1) declining informed consent; (2) not being a current student at the University of Milan (technical restriction via university email login ensured compliance); (3) incomplete questionnaires, defined as >20% of mandatory items left unanswered, including key demographic variables [35].

### 2.3. Data Collection

An anonymous, self-completed online survey was distributed via the university’s institutional email system. The questionnaire explored socio-demographic information, Mediterranean Diet adherence, and the use of food-related applications. Developed with Microsoft 365 A5 (Microsoft Corporation, Redmond, WA, USA), the survey was accessible exclusively to students with university credentials to restrict responses to the intended population. Duplicate entries were minimized through technical controls, and to enhance data completeness, all questions were mandatory, preventing advancement without a response. Participants were first provided with a Participant Information Sheet detailing the study’s objectives, procedures, potential risks, and benefits, and were required to provide written informed consent before proceeding. Non-consenting individuals were redirected to the end of the survey. The original documents were prepared in Italian; an English translation of the Participant Information Sheet and Informed Consent Form is available in Supplementary File S1.

### 2.4. Socio-Demographic and Behavioral Variables

The first section of the survey gathered information on participant characteristics, including socio-demographic variables: gender (female, male, prefer not to say), age, geographical area (North, Center-South, defined based on the geographical distribution of ISTAT (Istituto Nazionale di Statistica—National Institute of Statistics) in macro areas: North, including Piedmont, Valle D’Aosta, Liguria, Lombardy, Trentino-Alto Adige, Veneto, Friuli-Venezia Giulia and Emilia-Romagna, Center-South, including Tuscany, Umbria, Marche, Lazio, Abruzzo, Molise, Campania, Apulia, Basilicata, Calabria, Sardinia and Sicily), education level (High school diploma, Bachelor’s degree, Master’s degree or higher), number of family members, type of program enrolled (Bachelor’s degree, Master’s degree, Postgraduate degree or PhD), studying area (health sciences, humanities, science and technology, or social sciences), student status (commuter, out-of-town student, or residing in Milan), cohabitation (alone, with family, with a partner, or with friends/roommates). In addition, two behavioral variables were included based on prior evidence of their potential association with Mediterranean Diet adherence: smoking status (yes/no) and usage of food-related mobile applications (e.g., for delivery or surplus food saving). Smoking was considered because it frequently clusters with other unhealthy lifestyle behaviors, including poor dietary patterns, while the use of food-related mobile applications may influence food choices, either promoting less healthy options through food delivery services or encouraging sustainable behaviors through food waste reduction apps.

### 2.5. Mediterranean Diet Adherence

Mediterranea Diet adherence was assessed using the Medi-Lite questionnaire, a validated tool composed of nine items designed to evaluate adherence to the Mediterranean diet [36]. This instrument examines daily consumption of key food groups such as fruits, vegetables, cereals, and fish, assigning a total score that ranges from 0 (indicating low adherence) to 18 (indicating high adherence). In line with previous publications employing this index in European and non-European cohorts [37,38,39], a score of 12 or above was used to define high adherence to the Mediterranean dietary pattern.

### 2.6. Statistical Analysis

Descriptive statistics were used to summarize the socio-demographic and behavioral data and Mediterranean Diet, with means and standard deviations reported for continuous variables, and frequencies and percentages for categorical ones. Group differences in socio-demographic characteristics and questionnaire responses between levels of adherence to the Mediterranean diet were analyzed using the Welch *t*-test for continuous variables and either the Chi-square test or Fisher’s Exact Test for categorical variables, depending on the data distribution. Normality of the item responses was assessed using the Kolmogorov–Smirnov test, which indicated non-normal distribution across all items. To explore the relationship between socio-demographic data and Mediterranean Diet, Pearson’s correlation coefficient (r) was calculated. Logistic regression analyses were conducted to estimate odds ratios (ORs) and corresponding 95% confidence intervals (CIs) for the association between socio-demographic data and adherence to the Mediterranean Diet, with models adjusted for age and gender. Additional logistic models were used to assess the associations between food-related mobile applications and Mediterranean Diet adherence. Sensitivity analyses were conducted stratifying by gender and adjusting the model by age and educational level. A *p*-value of less than 0.05 was considered statistically significant.

A principal component analysis (PCA) was conducted to explore potential latent structures underlying the association between sociodemographic variables and adherence to the Mediterranean diet. The analysis included the following variables: age (continuous), gender (coded as 0 = male, 1 = female), education level (coded as 1 = higher education, 0 = otherwise), cohabitation status (dummy-coded: living alone, with partner, with family, or with roommates), geographical area of residence (dummy-coded: North, South/Islands, or abroad), and adherence to the Mediterranean diet, operationalized through nine food group–specific components: fruit, vegetables, legumes, cereals, fish, meat, dairy products, olive oil, and alcohol consumption. Prior to conducting principal component analysis (PCA), all variables were standardized to z-scores (mean = 0, standard deviation = 1) to ensure comparability and prevent scale-related biases in component extraction. PCA was performed using the correlation matrix of the standardized variables. The number of components retained was based on the proportion of variance explained and visual inspection of the scree plot. A biplot was constructed to display the distribution of individual participants along the first two principal components, with loading vectors overlaid to represent the magnitude and direction of each variable’s contribution. Variables were retained for graphical representation if the Euclidean norm of their loadings on PC1 and PC2 was ≥0.30. A heatmap of variable loadings was also generated using the same criterion, allowing for consistent interpretation of variable contributions across visualizations. All analyses were conducted using Stata/SE 18.5 (StataCorp LLC, College Station, TX, USA).

### 2.7. Sample Size Calculation

To estimate the required sample size, a 95% confidence level and a 5% margin of error were applied. The study population consisted of 60,988 students enrolled at the University of Milan during the academic year 2021/2022 [40]. A conservative estimate of 50% was used for the expected proportion, as this yields the largest possible sample size in proportion-based calculations. Under these conditions, the minimum number of participants required for the study was calculated to be 382.

## 3. Results

A total of 2779 responses were initially collected. Of these, 54 participants were excluded due to lack of informed consent, and an additional 28 were excluded due to missing data on the Medi-Lite score. As a result, the final analytical sample consisted of 2697 individuals (Supplementary Figure S1).

### 3.1. Socio-Demographic and Behavioral Characteristics According to Mediterranean Diet Adherence

Table 1 reports the socio-demographic and behavioral characteristics of students stratified by level of adherence to the MD. Among the total sample, the majority (*n* = 1941 students; 71.97%) showed low adherence (Medi-Lite score 0–11), and 756 (28.03%) showed high adherence (Medi-Lite score ≥ 12).

A significantly higher proportion of students in the high adherence group were women (74.2%), older (56.9%), with a higher level of education (25.9%), and living in smaller households (1–3 members).

Moreover, a lower proportion of smokers was observed in the high adherence group (24.9%) compared to the low adherence group (30.0%, *p* = 0.008), and among those living with a partner. Patterns of app usage differed significantly between groups, with students in the high adherence group were more likely to report never or rarely using food delivery apps (*p* < 0.001), and reported slightly higher use of food waste reduction apps (*p* = 0.004).

As expected, the mean Medi-Lite score was significantly higher in the high adherence group (12.83 ± 0.97) compared to the low adherence group (9.28 ± 1.61, *p* < 0.001).

### 3.2. Gender Differences in Mediterranean Diet Adherence Among University Students

**Figure 1:**
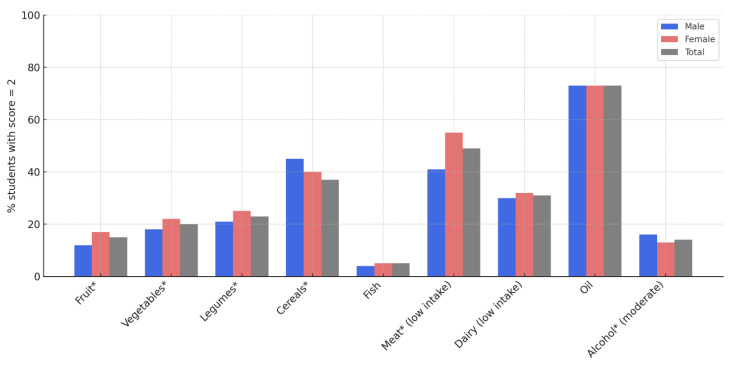
Proportion of university students achieving maximum (score = 2) adherence to individual mediterranean diet components, stratified by gender. Asterisks (*) indicate statistically significant gender differences (*p* < 0.05).

The distribution of students achieving the maximum score (2 points) for each component of the MD, stratified by gender, revealed notable differences across specific food groups (Figure 1).

The highest adherence was observed for olive oil consumption (≈70% in both genders), followed by cereals, with males scoring slightly higher. Women were significantly more likely than men to report high consumption of fruit, vegetables, legumes, and lower meat intake (*p* < 0.05). Fish and moderate alcohol intake showed the lowest adherence rates overall.

**Figure 2:**
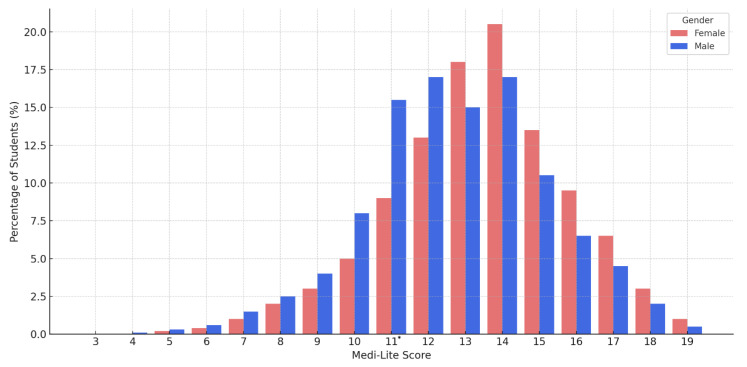
Distribution of Mediterranean Diet adherence scores (Medi-Lite) among university students, stratified by gender (male vs. female); asterisks indicate statistically significant differences between groups (Chi-Square Test, *p*  <  0.05).

The distribution of Medi-Lite scores among university students, stratified by gender, exhibited a near-normal pattern in both groups. Among females, the highest proportion of students was observed at score 14, while among males, peaks occurred at scores 12 and 14. A statistically significant difference between genders was identified only at score 11 (Figure 2).

### 3.3. Multivariable Regression Analysis of Socio-Demographic Factors Associated with Adherence to the Mediterranean Diet

As shown in Table 2, female students were significantly more likely to have high adherence to the MD, with a corresponding positive linear association. Age was also positively associated with adherence: students aged 24–29 and those aged ≥ 30 had higher odds of high adherence compared to those under 24 years, a trend mirrored in the linear regression model. Students residing in Central/Southern Italy were more likely to report high adherence compared to those in the North, with a significant positive association. Higher educational attainment was positively but not significantly associated with high adherence, while smoking showed a borderline inverse association. Student status also influenced outcomes: commuters had significantly lower odds of high adherence compared to residents in Milan, whereas off-site students and Erasmus students did not differ significantly. Living with a partner (vs. alone) was associated with significantly greater adherence, also confirmed in the linear model. Living with family or with friends/roommates did not show statistically significant associations. Use of digital applications also played a notable role: use of food waste apps was associated with higher odds of high adherence, while use of food delivery apps was associated with lower odds. Finally, the field of study and number of family members were not significantly associated with the outcome in either model.

### 3.4. Gender-Stratified Linear Regression Analysis

**Figure 3:**
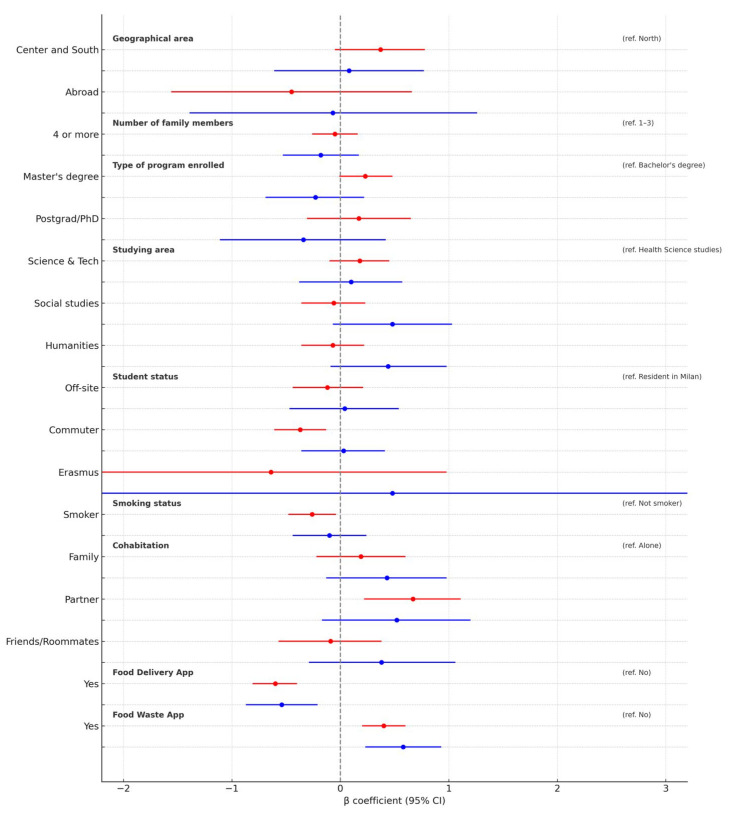
Forest plot showing the associations (β coefficients with 95% confidence intervals) between sociodemographic and behavioral characteristics and Mediterranean diet adherence score, stratified by gender. Estimates are adjusted for age and educational level. Women are shown in red, men in blue. Ref: reference.

As shown in Figure 3, gender-stratified models revealed notable differences in the factors associated with adherence to the MD. Among female students, smoking was negatively associated with adherence (β = −0.26; 95%CI: −0.48 to −0.04; *p* = 0.019), while living with a partner was positively associated with it (β = 0.67; 95%CI: 0.22 to 1.11; *p* = 0.003). For males, these associations were weaker and not statistically significant.

Both women and men who reported using food waste apps showed higher adherence scores (β = 0.40 and 0.58, respectively; *p* < 0.001 for both), while use of food delivery apps was inversely associated with adherence in both genders (β = −0.60 for women; β = −0.54 for men).

In terms of geographical area, the positive association with living in Central/Southern Italy was more evident among women (β = 0.37; *p* = 0.086), although not statistically significant, and absent among men. Among female students, being a commuter was negatively associated with adherence (β = −0.37; 95%CI: −0.61 to −0.13; *p* = 0.002), while this pattern was not observed in males.

These findings suggest that some determinants of dietary adherence may operate differently by gender, underlining the importance of stratified analyses. Full results are reported in Supplementary Table S1.

### 3.5. Principal Component Analysis (PCA) of Dietary Patterns and Sociodemographic Profiles

**Figure 4:**
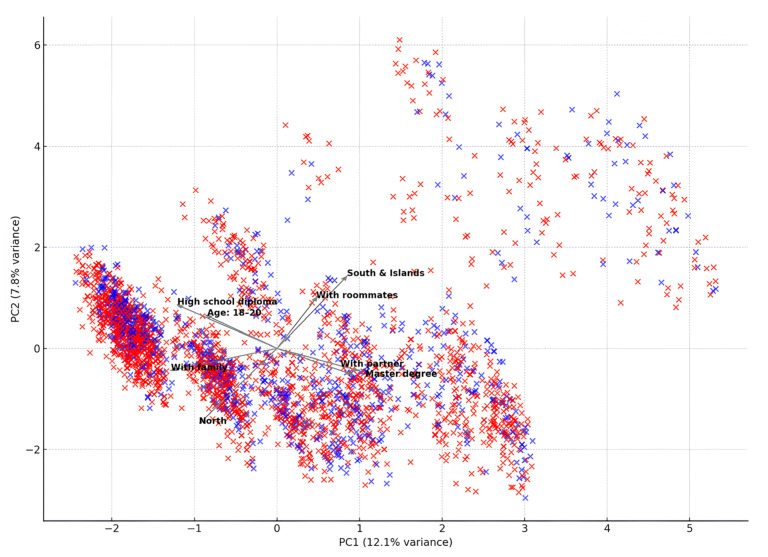
Biplot of the first two principal components derived from nine dietary components of the Mediterranean diet and sociodemographic variables. Each cross represents an individual participant, colored by gender (blue: male, red: female). Arrows indicate the direction and magnitude (loadings) of each variable’s contribution to the first (PC1) and second (PC2) principal components. Variables include dietary scores for fruit, vegetables, legumes, cereals, fish, meat, dairy products, olive oil, and alcohol; gender (dummy-coded), age category, educational level, cohabitation status, and geographic area (dummy-coded). The biplot illustrates the underlying structure and co-variation between sociodemographic profiles and adherence to the Mediterranean diet.

To explore underlying patterns in dietary behaviors and their relationship with sociodemographic characteristics, a principal component analysis (PCA) was conducted using the nine food-group components of the MD alongside selected demographic variables. The resulting biplot (Figure 4) provides a visual representation of individual distributions along the first two principal components (PC1 and PC2), which explained 12.1% and 7.8% of the total variance, respectively.

PC1 appears to capture a gradient of adherence to the MD, with individuals positioned on the right exhibiting higher scores across dietary components such as fruits, vegetables, legumes, fish, and olive oil, and lower consumption of red meat and alcohol. Conversely, participants located on the left side of the graph represent dietary profiles less aligned with Mediterranean dietary principles.

PC2 reflects variation primarily associated with sociodemographic attributes. Participants in the upper part of the plot are characterized by younger age, cohabitation with roommates, and residence in Southern Italy or the Islands. In contrast, the lower area of the graph is populated by individuals who are older, more likely to live with a partner or family, and to possess a higher level of education.

The joint interpretation of PC1 and PC2 indicates that individuals with greater adherence to the MD (right side) also tend to be older, more educated, and more often cohabiting or residing in Southern regions, whereas those with lower adherence (left side) are typically younger, live with family, and reside in Northern Italy. These results reinforce the presence of a socio-cultural and geographical gradient in dietary patterns, in line with findings from regression analyses.

**Figure 5:**
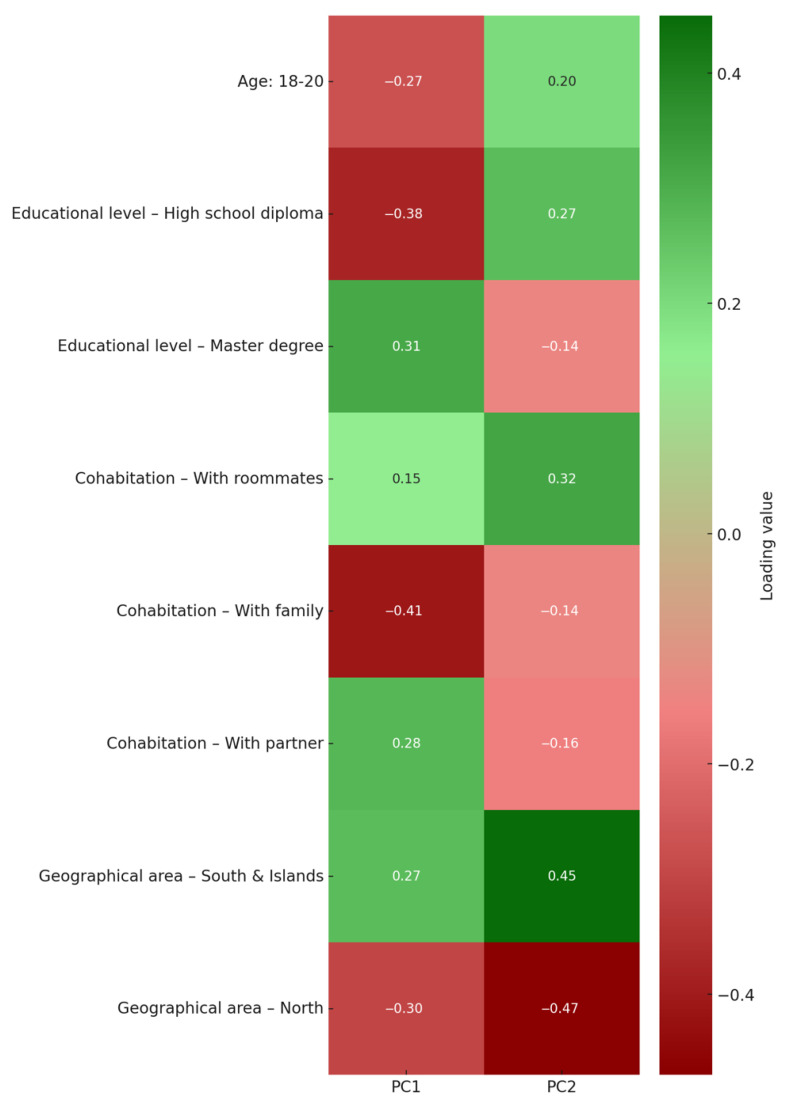
The heatmap displays the standardized loadings of sociodemographic variables and specific food group components contributing to Mediterranean diet adherence on the first (PC1) and second (PC2) principal components derived from principal component analysis (PCA). Variables were included if the Euclidean norm of their loadings across PC1 and PC2 was ≥ 0.30. Shades of red indicate negative contributions, while shades of green represent positive contributions. Darker colors correspond to stronger associations with the components, facilitating interpretation of the latent structure underlying dietary adherence and sociodemographic profiles.

To further elucidate the structure of the principal components, a heatmap was generated to visualize the standardized loadings of selected sociodemographic variables across PC1 and PC2 (Figure 5). Variables were included if their combined contribution (Euclidean norm) to the two components exceeded 0.30, highlighting the most influential features in shaping the latent structure.

Cohabitation with family and residence in northern regions displayed the strongest negative loadings on PC1 and PC2, suggesting an association with lower Mediterranean diet adherence and lower autonomy. Conversely, living in the South and Islands, cohabiting with roommates, and having a master’s degree exhibited strong positive loadings, particularly on PC2, indicating a profile characterized by higher independence and stronger adherence patterns. Notably, the loading pattern reinforces the interpretation that PC1 primarily captures dietary adherence, while PC2 reflects a sociodemographic gradient associated with autonomy, education, and geographic context.

These loading patterns offer a complementary perspective to the biplot representation, quantitatively supporting the observed clustering of individual characteristics and providing further insight into the multidimensional nature of dietary adherence.

## 4. Discussion

### 4.1. Interpretation of the Main Results

This study provides a comprehensive analysis of the socio-demographic and behavioral determinants associated with adherence to the Mediterranean diet among a large cohort of university students. Overall, our findings indicate that adherence to the Mediterranean dietary pattern remains suboptimal in this population, with only 28% of students reaching a high Medi-Lite score (≥12). When looking at the food groups, the findings suggest that while certain components of the Mediterranean diet—particularly the use of olive oil and the consumption of cereals—are well integrated into the dietary habits of students, substantial gaps remain in the intake of fish and in the consumption of fruits and vegetables.

Multivariable regression analyses revealed several socio-demographic factors significantly associated with higher adherence. Female students demonstrated a greater likelihood of following a Mediterranean dietary pattern compared to their male counterparts, a finding consistent with prior research suggesting gender-based differences in dietary behaviors and health awareness [41]. Our findings regarding gender differences in adherence to the Mediterranean diet are consistent with previous research showing that women tend to achieve higher adherence scores compared to men. This pattern has been partly attributed to greater health consciousness, better nutritional knowledge, and more positive attitudes toward healthy foods among women [42]. In addition, evidence from intervention studies suggests that women may have higher intrinsic motivation toward healthy eating, greater familiarity with plant-based foods, and more developed cooking skills, as well as differing social roles and responsibilities in meal preparation, all of which may contribute to their higher adherence to the Mediterranean dietary pattern [43]. Age was also positively associated with adherence, with older students (≥24 years) more likely to report healthier dietary patterns. This may reflect increased autonomy in food choices and greater nutritional awareness acquired over time [44].

Educational attainment showed a positive, albeit borderline significant, association with diet adherence, particularly for those holding a bachelor’s degree. Students residing in Central and Southern Italy had higher odds of adhering to the Mediterranean diet than those in the North, aligning with regional dietary traditions and cultural food practices. Notably, cohabitation with a partner was strongly associated with better adherence, potentially reflecting shared mealtime routines and more structured dietary habits.

Behavioral variables emerged as having important correlations with diet quality. Students who used food delivery apps exhibited significantly lower adherence, highlighting the detrimental impact of convenience-oriented food choices on nutritional quality. This relationship appears to be independent of age, sex, and education level, suggesting that the digital food environment may promote less healthy dietary behaviors through greater exposure to ultra-processed options and targeted marketing strategies [45]. In contrast, those who reported using food waste reduction apps were more likely to adhere to the Mediterranean diet, suggesting a possible link between sustainable food habits and healthier eating patterns [46]. Smoking status was negatively associated with dietary adherence, consistent with previous evidence showing clustering of unhealthy behavioral characteristics [47].

Importantly, PCA illustrated distinct co-variation patterns between dietary components and socio-demographic profiles, confirming the multivariate structure of Mediterranean diet adherence in this population. The observed clustering of female gender, older age, higher education, and use of sustainability-oriented apps along the same vector as key Mediterranean foods (e.g., fruits, vegetables, legumes, and olive oil) reinforces the role of these factors in shaping dietary patterns.

### 4.2. Public Health Implications

The results of this study have several important implications for public health strategies aimed at improving dietary behaviors among university students. The low proportion of students achieving high adherence to the MD is a cause for concern, particularly given the well-established benefits of this dietary pattern in the prevention of non-communicable diseases and the promotion of overall well-being [48]. University students represent a critical demographic group, as this life stage is characterized by the establishment of long-term health conduct and behavioral characteristics [49,50]. Our findings suggest the need for targeted interventions that address specific socio-demographic and behavioral risk factors. Given the associations between socio-demographic factors such as gender, age, education, and living arrangements with MD adherence, public health initiatives should prioritize engaging male students, younger age groups, and those with lower educational levels, as these individuals are less likely to adhere to the MD. In this context, university settings represent a valuable platform for implementing structured culinary and nutrition literacy interventions, including practical components such as cooking classes, digital tools for meal planning, and campus-wide health promotion campaigns [51,52]. Tailored interventions that take into account these demographic factors can increase the effectiveness of dietary promotion programs.

Moreover, the observed positive association between the use of food waste apps and higher dietary adherence highlights the potential for integrated interventions that promote both nutritional quality and environmental sustainability [53,54]. Actually, integrating sustainability education and fostering supportive social contexts may offer additional leverage points for improving dietary adherence in this vulnerable population [55]. These campaigns should be supplemented with information about the environmental benefits of adhering to sustainable food practices, as evidenced by the positive association between food waste reduction app usage and dietary adherence [56]. Furthermore, the negative impact of food delivery services on diet quality points to the need for public health campaigns that raise awareness about the long-term health risks associated with convenience foods [57,58]. The observed relationship between smoking and lower adherence to the Mediterranean diet further highlights the importance of addressing multiple behavioral characteristics simultaneously [59]. Public health programs that integrate smoking cessation with dietary interventions can help reinforce the adoption of healthier habits among university students [60,61]. Such programs may be more effective if they promote the interconnectedness of physical health, environmental sustainability, and long-term well-being [62,63].

Lastly, the role of cohabitation in supporting healthier dietary behaviors emphasizes the potential of social networks in shaping food choices [64,65]. Public health strategies that encourage communal eating and social cooking activities could reinforce the social and cultural dimensions of healthy eating, thereby improving dietary adherence [64,66].

In conclusion, public health initiatives aimed at improving adherence to the Mediterranean diet should adopt a holistic, multi-pronged approach that targets socio-demographic, behavioral, and social factors. By promoting sustainable and healthy eating practices, these interventions can play a key role in mitigating the rising prevalence of diet-related non-communicable diseases among young adults.

### 4.3. Strengths and Limitations

This study presents several strengths that enhance the validity and relevance of its findings. First, it draws on a large and diverse sample of university students (*n* = 2697), allowing for robust statistical power and a consistent analysis of socio-demographic and behavioral determinants of dietary adherence. Second, the use of the validated Medi-Lite score to assess adherence to the Mediterranean diet provides a standardized and reliable measure, facilitating comparability with other studies in similar populations [67]. Third, the multivariable approach—employing both logistic and linear regression models adjusted for key confounders—offers a comprehensive examination of the independent associations between predictors and dietary adherence [68]. Finally, the inclusion of PCA provides additional insight into the complex multivariate relationships between dietary components and socio-demographic characteristics, offering a more holistic understanding of adherence patterns [69].

However, several limitations should be acknowledged. The cross-sectional design of the study precludes the ability to establish causal relationships between socio-demographic or behavioral variables and adherence to the Mediterranean diet [70]. Additionally, data were collected through self-administered online questionnaires, which may be subject to recall bias, non-response bias or social desirability bias [71], particularly in the reporting of dietary intake and behavioral characteristics. However, we used an online, anonymous questionnaire, which has been consistently associated with lower risk of social desirability bias [72]. Some potential confounders, such as economic status and food literacy, were not assessed in our survey and therefore could not be included in the statistical models. These factors have been shown to influence dietary patterns and could be explored in future studies. Moreover, while the Medi-Lite score is validated, it provides a simplified representation of a complex dietary pattern and does not capture all dimensions of dietary quality or cultural adherence [73]. Furthermore, although the sample included students from diverse geographical origins, they were enrolled at a single university in Northern Italy, which may limit the generalizability of the findings to other academic or cultural contexts. Moreover, some potentially relevant factors—such as economic status and food literacy—were not assessed and may have influenced both diet quality and behavioral characteristics. Lastly, it is important to acknowledge certain methodological constraints related to the PCA. While the PCA provided valuable insights into latent structures linking dietary components and socio-demographic characteristics [74], the first two principal components explained only a limited proportion of the total variance (approximately 20%) [75]. This modest cumulative variance is in line with what is commonly observed in dietary and behavioral data [76], where high inter-individual variability and multidimensionality often result in dispersed component loadings. Nevertheless, it implies that the two-dimensional biplot offers a simplified representation of the data structure and may not fully capture the complexity of the underlying latent dimensions of MD adherence. Therefore, the PCA results should be interpreted as exploratory in nature, intended to facilitate visualization and pattern recognition [77]. While the two-component solution enhances interpretability and graphical clarity, particularly in the biplot format, it necessarily sacrifices a portion of the total explained variance and does not fully represent the multidimensional complexity of students’ dietary patterns and socio-demographic profiles [78].

### 4.4. Future Directions

Future research should prioritize longitudinal designs to establish causal relationships between socio-demographic, behavioral, and environmental factors and adherence to the MD among university students. Prospective cohort studies could help identify critical periods and life transitions that most strongly influence dietary behaviors. Additionally, future studies should incorporate a broader range of potential determinants, such as economic status, food literacy, psychosocial variables, and digital engagement, to develop a more comprehensive understanding of the drivers of MD adherence [79]. Given the emerging association between sustainable practices and healthier dietary patterns observed in this study, mixed-methods research exploring the motivations, barriers, and perceptions surrounding food sustainability among youth could provide valuable contextual insights [80,81]. Furthermore, interventional studies testing the effectiveness of integrated educational programs—combining nutrition literacy, culinary skills, and environmental sustainability—are warranted, particularly within university settings [82,83,84]. These programs should be tailored to subpopulations identified as at-risk, such as younger males and students with low educational attainment or high reliance on food delivery services. Finally, applying more sophisticated data-driven approaches could enhance the understanding of complex, multidimensional relationships among determinants of dietary adherence, thereby improving the precision of future public health interventions [85].

## 5. Conclusions

In this large sample of university students, adherence to the Mediterranean diet was generally low and influenced by a combination of socio-demographic and behavioral factors. Female gender, older age, higher education, cohabitation with a partner, and sustainable food practices were positively associated with better adherence, while use of food delivery services and smoking were negatively associated. These findings highlight the need for targeted, multifaceted public health strategies to promote healthier and more sustainable dietary behaviors among young adults in academic settings.

## Figures and Tables

**Table 1:** Socio-demographic and Behavioral characteristics of university students according to mediterranean diet adherence levels (low vs. high, based on Medi-Lite score).

	Low Adherence to MD (Medi-Lite: 0–11) *N* = 1941 (71.97%)	High Adherence to MD (Medi-Lite: ≥12) *N* = 756 (28.03%)	*p*-Value
Gender			0.031
Women	1341 (69.1)	561 (74.2)	
Men	566 (29.2)	185 (24.5)	
Prefer not to say	34 (1.8)	10 (1.3)	
Age			<0.001
18–23	1088 (56.1)	326 (43.1)	
24–29	570 (29.4)	287 (38.0)	
≥30	283 (14.6)	143 (18.9)	
Geographical area			0.093
North	1801 (92.8)	684 (90.5)	
Center and South	121 (6.2)	65 (8.6)	
Abroad	19 (1.0)	7 (0.9)	
Educational level			<0.001
High school diploma	1078 (55.5)	339 (44.8)	
Bachelor’s degree	470 (24.2)	221 (29.2)	
Master’s degree or higher	393 (20.3)	196 (25.9)	
Number of family members			0.023
1–3	747 (38.5)	327 (43.3)	
4 or more	1194 (61.5)	429 (56.8)	
Type of program enrolled			<0.001
Bachelor’s degree	962 (49.6)	313 (41.4)	
Master’s degree	751 (38.7)	331 (43.8)	
Postgraduate degree or Ph.D.	228 (11.8)	112 (14.8)	
Study area			0.066
Health Science studies	445 (22.9)	171 (22.6)	
Science and Technology studies	655 (33.8)	289(38.2)	
Social studies	371 (19.1)	117 (15.5)	
Humanities studies	470 (24.2)	179 (23.7)	
Student status			0.103
Resident in Milan	487 (25.1)	211 (27.9)	
Off-site	413 (21.3)	179 (23.7)	
Commuter	1035 (53.3)	363 (48.0)	
Erasmus	6 (0.3)	3 (0.4)	
Smoking status			0.008
Not smoker	1359 (70.0)	568 (75.1)	
Smoker	582 (30.0)	188 (24.9)	
Cohabitation			<0.001
Alone	175 (9.0)	55 (7.3)	
Family	1353 (69.7)	492 (65.1)	
Partner	198 (10.2)	128 (16.9)	
Friend(s) or Room-ate(s)	215 (11.1)	81 (10.7)	
Food Delivery App			<0.001
No	1060 (54.6)	487 (64.4)	
Rarely	877 (45.2)	269 (35.6)	
At least once a week	4 (0.2)	0 (0.0)	
Food Waste App			0.004
No	1266 (65.2)	446 (59.0)	
Rarely	670 (34.5)	305 (40.3)	
At least once a week	5 (0.3)	5 (0.7)	
Medi-Lite Score (mean and standard deviation)	9.28 (1.61)	12.83 (0.97)	<0.001

**Table 2:** Association between socio-demographic characteristics and high adherence to the MD (Medi-Lite ≥ 12). Multivariable logistic regression models adjusted for gender and age (n = 2697).

	αOR (95%CI); *p*-Value	β (95%CI); *p*-Value
Gender (ref. Male)		
Female	1.32 (1.08–1.61); 0.006	0.28 (0.08, 0.48); 0.006
Age group (ref. < 24)		
24–29	1.45 (1.15–1.82); 0.001	0.37 (0.14, 0.60); 0.001
≥30	1.38 (1.03–1.86); 0.032	0.32 (0.03, 0.62); 0.032
Geographical area (ref. North)		
Center and South	1.46 (1.02–2.09); 0.040	0.38 (0.02, 0.74); 0.040
Abroad	0.85 (0.35–2.09); 0.725	−0.16 (−1.06, 0.74); 0.725
Educational level (ref. High school diploma)		
Bachelor’s degree	1.24 (0.99–1.55); 0.055	0.22 (−0.00, 0.44); 0.055
Master’s degree or higher	1.11 (0.85–1.45); 0.440	0.11 (−0.16, 0.37); 0.440
Number of family members (ref. 1–3)		
4 or more	0.91 (0.75–1.10); 0.328	−0.09 (−0.28, 0.09); 0.328
Studying area (ref. Health Science studies)		
Science and Technology studies	1.15 (0.91–1.46); 0.248	0.14 (−0.10, 0.38); 0.248
Social studies	0.88 (0.67–1.17); 0.377	−0.13 (−0.41, 0.15); 0.377
Humanities studies	0.98 (0.76–1.26); 0.856	−0.02 (−0.28, 0.23); 0.856
Student status (ref. Resident in Milan)		
Off-site	1.01 (0.77–1.34); 0.925	0.01 (−0.27, 0.29); 0.925
Commuter	0.80 (0.65–0.99); 0.040	−0.22 (−0.44, −0.01); 0.040
Erasmus	0.94 (0.22–3.98); 0.933	−0.06 (−1.51, 1.38); 0.933
Smoking status (ref. Not smoker)		
Smoker	0.82 (0.68–1.00); 0.055	−0.19 (−0.39, 0.00); 0.055
Cohabitation (ref. Alone)		
Family	1.37 (0.96–1.95); 0.083	00.31 (−0.04, 0.67); 0.083
Partner	1.78 (1.21–2.63); 0.003	0.58 (0.19, 0.97); 0.003
Friend(s) or Roommate(s)	1.20 (0.79–1.81); 0.394	0.18 (−0.23, 0.59); 0.394
Food Delivery App (ref. No)		
yes	0.58 (0.48–0.69); <0.001	−0.55 (−0.74, −0.37); <0.001
Food Waste App (ref. No)		
yes	1.38 (1.15–1.66); 0.001	0.32 (0.14, 0.51); 0.001

αOR = adjusted Odds Ratio; CI = Confidence Interval, ref: reference.

## Data Availability

The data supporting the findings of this study are not publicly available due to ethical and privacy restrictions. The dataset contains sensitive information collected from human participants and cannot be shared openly to protect individual confidentiality. De-identified data may be made available by the corresponding author upon reasonable request and pending approval from the institutional ethics committee.

## Supplementary Materials

The following supporting information can be downloaded at: https://www.mdpi.com/article/10.3390/epidemiologia6030053/s1, Figure S1: Flow of participant selection; Table S1: Results of linear regression models estimating associations between sociodemographic and behavioral characteristics and Mediterranean diet adherence score, stratified by sex. The table presents β coefficients, 95% confidence intervals (CI), and *p*-values for women and men separately. All models were adjusted for age and educational level. Reference categories are indicated in parentheses; File S1: English translation of the Participant Information Sheet and Informed Consent Form used in the study. The original version, administered to participants, was in Italian.
