# Supplementary material for: The Relationship Between Socio-Demographic and Behavioral Characteristics and Adherence to the Mediterranean Diet: The UniFoodWaste Study Among University Students in Italy
Source: Epidemiologia (Basel). 2025 Sep 3;6(3):53. doi: 10.3390/epidemiologia6030053 (PMC12452464; doi:10.3390/epidemiologia6030053)
Supplement: Supplementary file 1 [file epidemiologia-06-00053-s001.zip › epidemiologia-3805153-supplementary.pdf]

Supplementary

The relationship between socio-demographic and behavioral characteristics and adherence to the Mediterranean diet: the UniFoodWaste study among university students in Italy

Figure S1. *Flow of participant selection.*

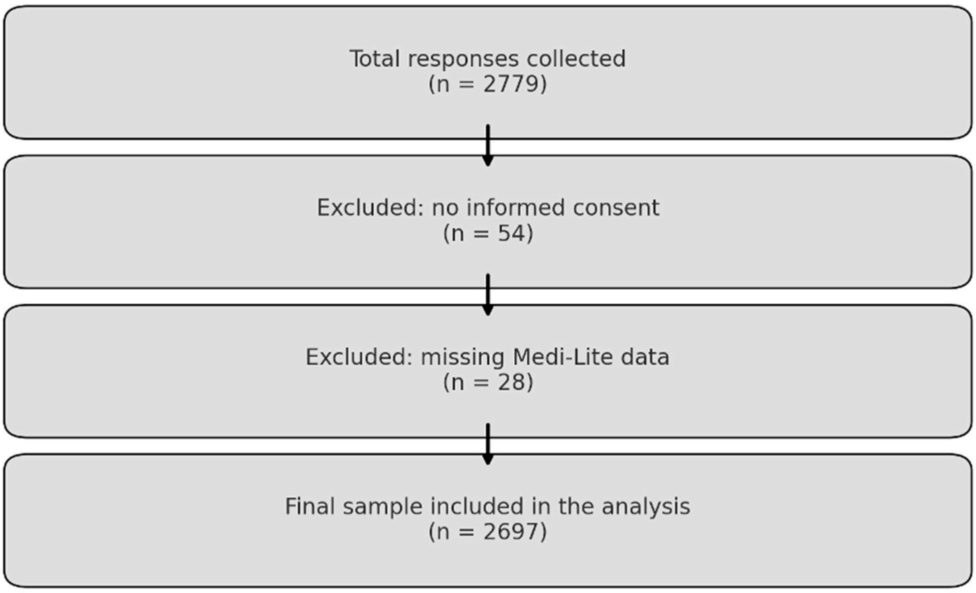

Table S1. Results of linear regression models estimating associations between sociodemographic and behavioral characteristics and Mediterranean diet adherence score, stratified by sex. The table presents β coefficients, 95% confidence intervals (CI), and p-values for women and men separately. All models were adjusted for age and educational level. Reference categories are indicated in parentheses.

	**Women β (95% CI); p**	**Men β (95% CI); p**
**Geographical area** (ref. North)		
Center and South	0.37 (–0.05, 0.78); 0.086	0.08 (–0.61, 0.77); 0.829
Abroad	–0.45 (–1.56, 0.66); 0.428	–0.07 (–1.39, 1.26); 0.922
**Number of family members** (ref. 1-3)		
4 or more	–0.05 (–0.26, 0.16); 0.645	–0.18 (–0.53, 0.17); 0.310
**Type of program enrolled** (ref. Bachelor's degree)		
Master's degree	0.23 (–0.01, 0.48); 0.064	–0.23 (–0.69, 0.22); 0.315
Postgraduate degree or PhD	0.17 (–0.31, 0.65); 0.486	–0.34 (–1.11, 0.42); 0.376
**Studying area** (ref. Health Science studies)		
Science and Technology studies	0.18 (–0.10, 0.45); 0.204	0.10 (–0.38, 0.57); 0.681
Social studies	–0.06 (–0.36, 0.23); 0.688	0.48 (–0.07, 1.03); 0.090
Humanities studies	–0.07 (–0.36, 0.22); 0.656	0.44 (–0.09, 0.98); 0.105
**Student status** (ref. Resident in Milan)		
Off-site	–0.12 (–0.44, 0.21); 0.474	0.04 (–0.47, 0.54); 0.883
Commuter	**–0.37 (–0.61, –0.13); 0.002**	0.03 (–0.36, 0.41); 0.895
Erasmus	–0.64 (–2.26, 0.98); 0.440	0.48 (–2.54, 3.49); 0.756
**Smoking status** (ref. Not smoker)		
Smoker	**–0.26 (–0.48, –0.04); 0.019**	–0.10 (–0.44, 0.24); 0.566
**Cohabitation** (ref. Alone)		
Family	0.19 (–0.22, 0.60); 0.366	0.43 (–0.13, 0.98); 0.132
Partner	**0.67 (0.22, 1.11); 0.003**	0.52 (–0.17, 1.20); 0.138
Friend(s) or Roommate(s)	–0.09 (–0.57, 0.38); 0.709	0.38 (–0.29, 1.06); 0.267
**Food Delivery App** (ref. No)		
Yes	**–0.60 (–0.81, –0.40);<0.001**	**–0.54 (–0.87, –0.21);0.001**
**Food Waste App** (ref. No)		
Yes	**0.40 (0.20, 0.60); <0.001**	**0.58 (0.23, 0.93); 0.001**

File S1. English translation of the Participant Information Sheet and Informed Consent Form used in the study. The original version, administered to participants, was in Italian.

UNIVERSITÀ DEGLI STUDI DI MILANO

INFORMATION SHEET

Domestic food waste. Fact-finding survey among university students

---

Dearest,
We want to propose that you participate in research. It is your right to be informed about the purpose and characteristics of the study so that you can decide consciously and freely whether to participate. We invite you to carefully read the following. The researchers involved in this project are available to answer your questions.

Data controller	Rector pro tempore<br>University of Milan Via<br>Festa del Perdono n. 7,<br>20122 Milan
University of Milan	(rector@unimi.it)
Data Protection Officer	Prof. Pierluigi Perri<br>pierluigi.perri@unimi.it
Data Protection Officer of University	(dpo@unimi.it)
Professor or researcher scientific contact of the study	Vincenza Gianfredi<br>vincenza.gianfredi@unimi.it

According to the Ministry of Health, food waste is defined as “the set of products discarded from the agri-food chain, which for economic, aesthetic reasons or due to the proximity of the consumption deadline, although still edible and therefore potentially intended for human consumption, are destined to be eliminated or disposed of”. Food losses occurred throughout the food supply chain, however approximately 61% of total waste globally is attributable to domestic consumption. In consideration of the strong environmental, economic and social impact that food waste has, and in light of the need to develop actions aimed at understanding and consequent combating food waste, this study intends to understand what the determinants of domestic food

waste are, and whether these are associated with our health. The determinants potentially associated with household food waste and that will be investigated in this study are related to lifestyles and include: physical activity performed, the tabagic habit, alcohol consumption and adherence to the Mediterranean diet. While, data relating to health such as body weight and height, perceived health status, the presence of eating disorders and depressive symptoms. Finally, socio-demographic data such as sex, age in categories, region of residence, level of education, student status, disciplinary area of the course of study. Your participation is completely free, refusal to participate will not entail any negative consequences. Furthermore, if you change your mind and wish to withdraw, you are free to do so at any time without having to provide any explanation. In the event of withdrawal, the data previously collected will be destroyed unless it has already been processed for research purposes.
Participation in the study takes place after detailed information on its characteristics, risks and benefits. At the end of the information phase you will be able to consent to participation in the study.
By signing the informed consent form. Only after you have expressed your consent in writing will you be able to actively participate in the proposed study.

The research project involves the compilation of a short and anonymous questionnaire, and the subsequent analysis of the data collected for the purposes indicated above.
The total duration of the project will be 12 months
His involvement in the project will be approximately 10 minutes. In particular, you will be asked to indicate, through closed-ended questions, some of your behaviors related to food waste, some lifestyles, and health outcomes (as detailed above).
All information relating to the processing of your personal data (including special categories of data) is contained in the appropriate information drawn up pursuant to Article 13 of the Reg. 2016/679 (GDPR) issued together with this information sheet.
The study does not entail risks or direct benefits for the participant. However, the study will allow us to increase knowledge regarding domestic food waste and its determinants.

study was approved by the Ethics Committee of the University of Milan.
The original of the written Informed Consent signed by you will be kept by the person responsible for this study, while you are entitled to receive a copy.
During the study, you can contact the study manager for any information.

Thank you for your availability

STATEMENT BY THE STUDY MANAGER

I declare that I have provided the participant with complete information and detailed explanations regarding the nature, purposes, procedures and duration of this research project. I also declare that I have provided the information sheet to the participant.

SIGNATURE OF THE PERSON RESPONSIBLE FOR THE STUDY   Data

Dott.ssa Vincenza Gianfredi

INFORMED CONSENT
I declare that, with the information sheet, I have received explanations about the research project, the risks and benefits arising from the study and have been informed of my right to withdraw at any time

- ◉ Yes
- ○ No

If he answers No →
Having selected the No option, you will not be able to continue filling out the questionnaire.
You can close the browser window to finish.
If the compiler selects I agree →

INFORMATION ON THE PROCESSING OF PERSONAL DATA
Click **here** to read the information on the processing of personal data

---

CONSENT TO THE PROCESSING OF PERSONAL DATA
I declare that I have read the information drawn up pursuant to Article 13 reg. EU 2016/679 and on the basis of what is contained therein

- ◉ I consent to the processing of personal data
- ○ I do not consent to the processing of personal data

If the compiler selects I do not agree →
Having selected the option "I do not consent to the processing of personal data", you will not be able to continue completing the questionnaire.
You can close the browser window to finish.
If the compiler selects I agree, he can proceed with completing the questionnaire
